# Construction of a Multifunctional Nano-Scale Metal-Organic Framework-Based Drug Delivery System for Targeted Cancer Therapy

**DOI:** 10.3390/pharmaceutics13111945

**Published:** 2021-11-17

**Authors:** Mengru Cai, Yawen Zeng, Manting Liu, Longtai You, Huating Huang, Yang Hao, Xingbin Yin, Changhai Qu, Jian Ni, Xiaoxv Dong

**Affiliations:** 1 School of Chinese Material Medica, Beijing University of Chinese Medicine, Beijing 102488, China; 20210941442@bucm.edu.cn (M.C.); 20180935124@bucm.edu.cn (Y.Z.); 20200935153@bucm.edu.cn (M.L.); 20190941307@bucm.edu.cn (L.Y.); 20210935123@bucm.edu.cn (H.H.); yxbtcm@bucm.edu.cn (X.Y.); quchanghai@bucm.edu.cn (C.Q.); 2 Department of Radiology, Division of Translational Nanobiomaterials and Imaging, Leiden University Medical Center, NL-2333 ZA Leiden, The Netherlands; Y.Hao@lumc.nl

**Keywords:** MOFs, targeting drug delivery, cancer, controlled release

## Abstract

The antitumor activity of triptolide (TP) has received widespread attention, although its toxicity severely limits its clinical application. Therefore, the design of a targeted drug delivery system (TDDS) has important application prospects in tumor treatment. Metal–organic frameworks (MOFs), with high drug-carrying capacity and good biocompatibility, have aroused widespread interest for drug delivery systems. Herein, folic acid (FA) and 5-carboxylic acid fluorescein (5-FAM) were used to modify Fe-MIL-101 to construct a functionalized nano-platform (5-FAM/FA/TP@Fe-MIL-101) for the targeted delivery of the anti-tumor drug triptolide and realize in vivo fluorescence imaging. Compared with Fe-MIL-101, functionalized nanoparticles not only showed better targeted therapy efficiency, but also reduced the systemic toxicity of triptolide. In addition, the modification of 5-FAM facilitated fluorescence imaging of the tumor site and realized the construction of an integrated nano-platform for fluorescence imaging and treatment. Both in vitro and in vivo studies of functionalized nanoparticles have demonstrated excellent fluorescence imaging and synergistic targeting anticancer activity with negligible systemic toxicity. The development of functional nano-platform provides new ideas for the design of MOF-based multifunctional nano-drug delivery system, which can be used for precise treatment of tumor.

## 1. Introduction

At present, most chemotherapeutic drugs have been proven to have good anti-tumor effects, but their uneven distribution in the body and side effects on normal tissues and organs often affect their clinical applications [1]. During treatment, an increase in dose is used to solve the problem of biodistribution, but it can cause dose-related side effects [2]. Therefore, there is an urgent need for a targeted drug delivery system with biocompatibility to overcome the above-mentioned problems [3]. The rapid development of nanotechnology provides an opportunity to overcome challenges faced by the biomedical field.

As a multifunctional porous nanomaterial, metal–organic frameworks (MOFs) have received extensive attention in various fields since they were proposed [4,5,6]. They have been widely applied in gas storage, adsorption, catalysis, sensing, biological imaging, energy storage, and drug delivery [7,8,9,10,11,12,13]. The adjustable structure of MOFs can achieve targeted drug delivery, which is currently a research hotspot in the field of biomedicine [14]. Zhang et al. utilized a folate-modified MOF as a carrier of photosensitizers to achieve precise tumor treatment [15]. Vandana et al. synthesized MIL-101-Fe with a solvothermal method and achieved drug coupling through amino groups. The nanoparticles were proven to have a good drug release profile [16]. Gao et al. modified ZIF-8 with folic acid and 5-FAM to target the delivery of 5-FU. This nano-platform exhibited targeted drug delivery, affecting tracking and local slow-release functions [17].

Among MOFs, iron-based MOFs have been studied extensively due to their excellent properties and chemical versatility [18,19,20,21]. Folic acid (FA) receptors are highly expressed in tumor tissues; therefore, metal–organic frameworks modified by folic acid can be used for tumor targeted drug delivery, which improves the uptake of drugs by tumor tissues and reduces systemic toxic and side effects [22,23,24,25]. As a common fluorescent agent, 5-carboxyluciferin (5-FAM) has widely been used as a fluorescent probe due to its good water solubility and high fluorescence quantum yield [26,27]. MOFs modified with 5-FAM can realize real-time fluorescence imaging in vivo.

Triptolide (TP), a diterpenoid compound, is one of the main bioactive components of the Chinese herb *Tripterygium wilfordii* Hook. F. [28]. It has a variety of pharmacological effects, such as being immunosuppressive, anti-inflammatory, and anti-cancer, but its poor water solubility and adverse reactions (mainly in the reproductive system, liver, kidney, etc.) seriously restrict its clinical applications [29,30,31,32,33,34]. The emergence of targeted drug delivery systems (TDDSs) can deliver drugs to tumor sites, reduce the accumulation of drugs in normal tissues, and thus greatly reduce their toxicity.

In order to enhance the anti-tumor effect of TP while reducing its toxicity, we designed and constructed a multifunctional nano-therapeutic drug with 5-FAM/FA/TP@Fe-MIL-101 NPs as nanocarriers, 5-FAM as a fluorescent imaging agent, and FA as a targeting ligand. We evaluated the efficacy and biological safety of the nano-platform in vivo and in vitro. The results showed that the antitumor efficacy of TP was obviously enhanced and its toxicity was reduced, which provided a new idea for the targeted delivery of chemotherapeutics. We envisage that this 5-FAM/FA/TP@Fe-MIL-101 will prove to be a novel drug delivery system for the treatment of cancer diseases. Through the comprehensive evaluation of Fe-MIL-101 for the delivery of anti-tumor drugs, we have proven the feasibility of 5-FAM/FA/TP@Fe-MIL-101 NPs as a new type of nano-platform, which may be used in a wide range of anti-tumor treatments.

## 2. Materials and Methods

### 2.1. Synthesis MOFs

#### 2.1.1. Synthesis of Fe-MIL-101

Fe-MIL-101 MOFs were synthesized using the solvothermal method, as previously reported [35,36]. The reaction conditions, including the reaction time, temperature, and the molar ratio of FeCl_3_·6H_2_O to NH_2_-H_2_BDC, were carefully optimized. Amounts of 2 mmol of FeCl_3_·6H_2_O and 1 mmol of 2-amino-terephthalic acid (NH_2_-H_2_BDC) were dissolved in 35 mL of DMF and then transferred to an autoclave and reacted at 110 °C for 24 h. After cooling to room temperature, the obtained precipitates were washed with DMF and vacuum-dried at 110 °C for 12 h.

#### 2.1.2. Synthesis of TP@Fe-MIL-101

A total of 10 mg Fe-MIL-101 was dispersed in 2 mL methanol solution; then, TP was added to the above solution. The mixture was stirred at room temperature for 72 h. After centrifugation, the obtained product was washed with methanol three times to obtain TP@Fe-MIL-101. The supernatant was collected, and the free TP concentration was determined by HPLC. The drug loading capacity of TP@Fe-MIL-101 is defined as follows: drug loading capacity (%) = weight of TP in TP@Fe-MIL-101/weight of TP@Fe-MIL-101 × 100%.

#### 2.1.3. Synthesis of 5-FAM/FA/TP@Fe-MIL-101

5-FAM/FA/TP@Fe-MIL-101 MOFs were prepared by post-synthesis modification. Masses of 10 mg of TP@Fe-MIL-101, 10 mg of FA, and 10 mg of 5-FAM were dissolved in 20 mL PBS solution (pH 7.4), followed by the addition of EDC (10 mg) and NHS (10 mg); then, the mixture was stirred for 20 h in the dark at room temperature. After the reaction, 5-FAM/FA/TP@Fe-MIL-101 MOFs were obtained by centrifugation.

### 2.2. Characterizations of NPs

The surface morphology of the NPs was investigated by high-resolution transmission electron microscopy (TEM, JEM-2100, JEOL Corporation, Tokyo, Japan) and field emission scanning electron microscopy (SEM, JSM-7500F, JEOL Corporation, Tokyo, Japan). X-ray diffraction (XRD) was recorded with a Rigaku Uitima IV X-ray diffractometer. Nitrogen adsorption–desorption isotherms were recorded with a nitrogen adsorption–desorption analyzer (Bel Japan Inc., Tokyo, Japan). The simulation atomic coordinates are freely available for download from the Cambridge Crystallographic Data Centre. The structures were evaluated with a Fourier-transform infrared spectrometer (FTIR, NICOLET 6700, Thermo Fisher, Waltham, MA, USA). Thermogravimetric analysis (TGA) was carried out on a Mettler-Toledo thermogravimetric analyzer under a nitrogen flow in the range of room temperature to 800 °C. A Malvin nanometer laser particle size analyzer (Zetasizer Nano S90, Malvern Company, Malvern, UK) was used to measure the particle size distribution and zeta potential.

### 2.3. In Vitro Drug Release Study

The drug-loaded NPs were placed in a dialysis bag (8–14 KD) with 50 mL PBS (pH 7.4 and pH 5.5) at 37 °C. At different time points, 1 mL of solution was taken out, and the same amount of fresh buffer was added. The TP concentration was determined by HPLC. The mobile phase was a mixture of methanol and water (50:50), the column temperature was 25 °C, the flow rate was 1.0 mL/min, and the detection wavelength was 218 nm. The cumulative release percentage of TP from 5-FAM/FA/TP@Fe-MIL-101 was calculated.

### 2.4. Cell Culture

A HepG2 cell line was purchased from Guangzhou Jenniobio Biotechnology Co., Ltd., Guangzhou, China, and HL-7702 cells were obtained from the China Infrastructure of Cell Line Resources. Cells were cultured in high-glucose DMEM containing 10% (*v*/*v*) fetal bovine serum and 1% (*v*/*v*) penicillin–streptomycin. The cells were then placed in an incubator at 37 °C with 5% CO_2_.

### 2.5. In Vitro Cytotoxicity Assay

The MTT method was used to evaluate the toxicity of Fe-MIL-101 on HL-7702 cells and the antitumor effect of drug-loaded NPs on HepG2 cells. Cells were inoculated into 96-well plates (7 × 10^3^ cells/well) and cultured in an incubator for 12 h. NPs with different concentrations (TP: 1, 0.5, 0.25, 0.125, 0.0625 µg/mL) were added. After a certain time, MTT was treated for another 4 h. Then, DMSO was added to dissolve the blue Formazan crystals by low-speed vibration for 5 min in a shaker. The absorbance was measured at 490 nm using a microplate analyzer (BioTek Instruments Inc., Winorsky, VT, USA).

### 2.6. Annexin V/PI Double-Staining Assay

HepG2 cells (4 × 10^5^ cells/mL) were seeded in six-well plates and incubated overnight; then, functionalized NPs of different concentrations (0.25, 0.5, 1 µg/mL TP) were incubated for 12 h. After 40 s of trypsin digestion, the cells were collected by centrifugation, washed with PBS, and resuspended with binding buffer (295 μL). Annexin V-FITC (5 µL) and PI (10 µL) were added and incubated at room temperature for 15 min in the dark. Cell apoptosis was detected by flow cytometry after filtration with a nylon mesh (300 mesh).

#### 2.6.1. Measurement of Intracellular ROS

HepG2 cells were treated with functionalized NPs (0.5 μg/mL TP) for 12 h, and 1 mL DCFH-DA (10 µM) was added and incubated at room temperature for 20 min in the dark. The cells were washed twice with PBS, and the intracellular reactive oxygen species (ROS) were observed under the excitation wavelength of 488 nm using an inverted fluorescence microscope (ECLIPSE Ts2R, NIKON Instrument Co., Ltd., Tokyo, Japan).

#### 2.6.2. Measurement of Mitochondrial Membrane Potential (MMP)

HepG2 cells were treated with drug-loaded NPs (0.5 μg/mL TP) for 12 h. Then, 1 mL of JC-1 (10 µM) was added and incubated at room temperature for 10 min in the dark. The cells were washed twice with PBS and observed under an inverted fluorescence microscope (ECLIPSE Ts2R, NIKON Instruments Co., Ltd).

### 2.7. Cell Uptake Study

Cells were seeded into six-well plates at a density of 4 × 10^5^ cells/well and incubated overnight. HepG2 cells were treated with 5-FAM/FA/TP@Fe-MIL-101 and 5-FAM/TP@Fe-MIL-101 (1 µg/mL TP) for 4 h, and then the old medium was discarded. The cells were washed with PBS three times and observed with a laser confocal microscope under an excitation wavelength of 480 nm.

### 2.8. Western Blot Analysis

Western blotting was used to detect the effect of TP on apoptotic proteins in HepG2 cells. TP and functionalized NPs (1 µg/mL TP) were used to treated cells for 12 h. Then, the treated cells were washed twice with cold PBS and lysed with RIPA buffer for 30 min. The lysate was centrifuged at 4 °C (12,000 r/min, 10 min), and the total protein concentration was determined using a BCA protein assay kit. The mitochondria and cytoplasm were isolated using the Proteoextract^®^Cytosol/Mitochondrial Isolation Kit. The protein lysates were separated by 10% SDS-PAGE and transferred to a PVDF membrane with a TBST buffer containing 5% skimmed milk. The membrane was sealed for 1 h and incubated overnight at 4 °C with primary antibody [CytC (1:5000), Bcl-2 (1:5000), Bax (1:2000), cleaved PARP (1:1000), cleaved Caspase 3 (1:500), cleaved Caspase 9 (1:2000), p53 (1:1000), COX-IV (1:5000), and β-actin (1:5000)]. Then, it was incubated with the horseradish peroxidase-conjugated secondary antibodies (1:5000 dilution) at room temperature for 1 h. The signal of the target protein was collected by an ECL detection system. All of the experimental results were repeated at least three times.

### 2.9. Animal and Tumor-Bearing Mouse Model

All the animal experiments were performed in accordance with the guidelines approved by the Institutional Animal Care and Use Committee of Beijing University of Chinese Medicine. Balb/c nude mice (8–10 weeks, 15 g) were obtained from Sipeifu Biological Technology Co., Ltd. (Beijing, China), and all animal care and handing procedures were in accordance with the guidelines approved by the ethics committee of Beijing University of Chinese Medicine. A tumor-bearing mouse model was established initially. HepG2 cells were suspended in saline and subcutaneously injected in the right anterior armpit posterior of mice (1 × 10^7^ cells/mL, 0.1 mL). Then, tumor volume was calculated as V = W^2^ × L/2.

### 2.10. Distribution of Functionalized Nanoparticles In Vivo

When the tumor volumes reached 150 mm^3^, the HepG2 tumor-bearing mice were divided into two groups (*n* = 5). 5-FAM/FA/TP@Fe-MIL-101 and 5-FAM/TP@Fe-MIL-101 (100 μg/mL) were administered through the tail vein. After isoflurane anesthesia, the fluorescence images were recorded with a small animal in vivo imaging system (Ex = 490 nm, Em = 520 nm).

### 2.11. Antitumor Effect of Functionalized Nanoparticles In Vivo

Fifteen tumor-bearing mice were randomly divided into three groups, and each group was injected with saline, TP (0.2 mg/kg), or functional NPs (a TP concentration of 0.2 mg/kg) through the tail vein every 48 h. The body weights, tumor size, and tumor weights of the mice were measured. After 12 days of treatment, tumors were collected, and then subjected to DAPI staining and TUNEL assays. The major organs of the mice were harvested for H&E staining on the 12th day to determine the toxicity of the NPs.

### 2.12. Safety Evaluation of Functionalized NPs In Vivo

Blood of C57BL/6 mice treated with NPs (a TP concentration of 0.2 mg/kg) for 14 d was collected, and the samples were used to carry out blood biochemical assays. Blood from the mice without any treatments was defined as the control. The liver, spleen, and kidney (right kidney) were collected and stained with hematoxylin–eosin (H&E) to investigate the biotoxicity.

### 2.13. Statistical Methods

All experiments were performed with at least three independent replications, and data were expressed as means ± SDs. Comparisons were analyzed using one-way variance analysis (ANOVA). * *p* < 0.05 and ** *p* < 0.01 were considered to be statistically significant.

## 3. Results

### 3.1. Preparation and Characterization of NPs

Fe-MIL-101 were synthesized using the solvothermal method [37]. As shown in Figure S1, the characteristic peaks of Fe-MIL-101 were obvious (2θ = 8.9°, 9.5°, 10.6°), and the crystallinity was good. Fe-MIL-101 NPs had a regular octahedral shape and monodisperse particles with a similar average size of 290.7 nm (Figure S2). In addition, the XRD spectra of the synthesized MOFs were in good agreement with the simulated images of Fe-MIL-101 (Figure S3a), indicating the successful synthesis of Fe-MIL-101. Simultaneously, N_2_ adsorption–desorption isotherms were obtained to check the porosity of Fe-MIL-101 (Figure S3b). The specific surface area of Fe-MIL-101 was 480.86 m^2^/g and the pore size was about 2.43 nm.

**Figure 1:**
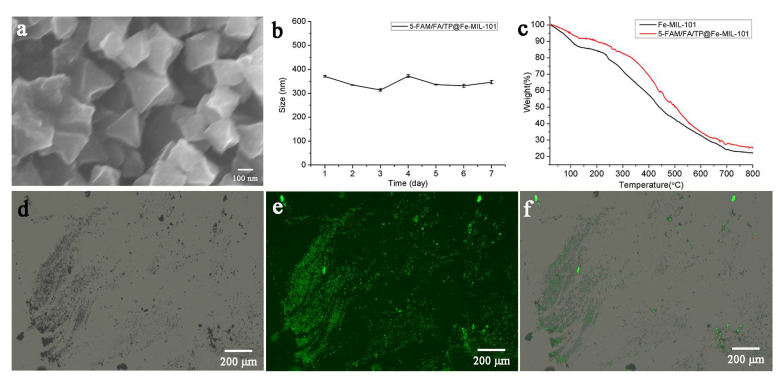
(**a**) SEM of 5-FAM/FA/TP@Fe-MIL-101; (**b**) the size of 5-FAM/FA/TP@Fe-MIL-101 within 7d (*n* = 3); (**c**) TGA curves of Fe-MIL-101 and 5-FAM/FA/TP@Fe-MIL-101; (**d**) bright-field image of 5-FAM/FA/TP@Fe-MIL-101; (**e**) fluorescence imaging of 5-FAM/FA/TP@Fe-MIL-101; (**f**) merge imaging of 5-FAM/FA/TP@Fe-MIL-101.

Additionally, TP was loaded on Fe-MIL-101 using the solvent adsorption method. In order to achieve active targeted drug delivery and fluorescence imaging, FAM/FA/TP@Fe-MIL-101 was prepared by post-synthesis modification [38,39,40]. The TP loading capacity in FAM/FA/TP@Fe-MIL-101 was 39.77 ± 1.24%. Scanning electron microscopy (SEM) showed that the morphology and size of NPs after modification did not change much (Figure 1a). The successful modification of FA and FAM was confirmed via the following experiments. The infrared spectrum of Fe-MIL-101 showed a characteristic peak of a N–H bond at 1575 cm^−1^ (Figure S4). The vibrational absorption peaks of carboxyl groups in FA and 5-FAM appeared at 1695 cm^−1^ and 1696 cm^−1^, respectively. The appearance of the characteristic peak of the amide group at 1607 cm^−1^ and the disappearance of the characteristic peak of the carbonyl group in 5-FAM/FA/TP@Fe-MIL-101 indicated that FA and 5-FAM successfully combined with –NH_2_ on the surface of the MOFs. In addition, FAM/FA/TP@Fe-MIL-101 had obvious green fluorescence from 5-FAM under a fluorescence microscope (Figure 1d–f). The particle size of functional NPs ranged from 300 to 400 nm, which was stable in PBS solution within 7 days (Figure 1b). When FA and 5-FAM were modified, the Zeta potential of FAM/FA/TP@Fe-MIL-101 changed from positive (Fe-MIL-101, 9.97 ± 1.02 mV; TP@Fe-MIL-101, 34.12 ± 0.76 mV) to negative (−13.14 ± 2.34 mV). According to the thermogravimetric curve (Figure 1c), there were two main weightlessness processes of Fe-MIL-101. The weight loss was about 15% at 30~150 °C, which indicated the weight loss of residual solvent and water molecules in the frame structure. In the range of 150~250 °C, the weight remained stable, whereas the skeleton of the NPs collapsed at 250~700 °C, indicating that the NPs exhibited good thermal stability within 250 °C. The TGA curves of 5-FAM/FA/TP@Fe-MIL-101 were not completely consistent with those of Fe-MIL-101, suggesting that the decomposition of 5-FAM/FA/TP@Fe-MIL-101 was accompanied by the decomposition of FA and 5-FAM.

### 3.2. Study on the Drug Release of Functionalized Nanoparticles In Vitro

**Figure 2:**
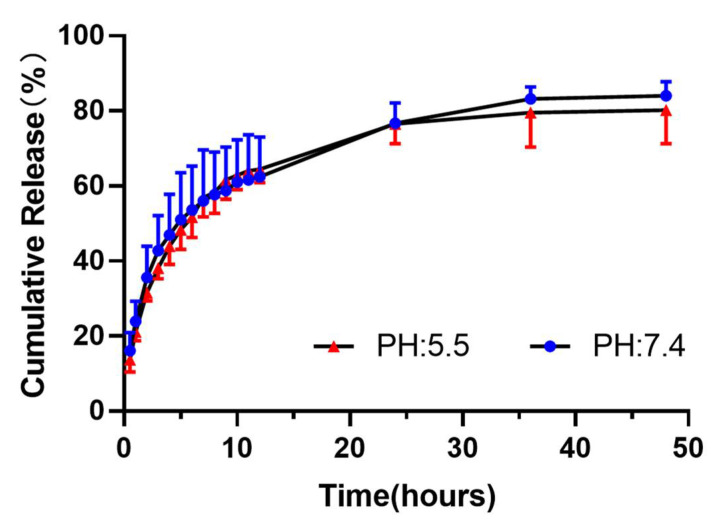
Drug release curve of 5-FAM/FA/TP@Fe-MIL-101 in PBS at pH 7.4 and 5.5 (*n* = 3).

The pH-responsive release of TP from 5-FAM/FA/TP@Fe-MIL-101 NPs was investigated. The data demonstrated that 5-FAM/FA/TP@Fe-MIL-101 NPs had similar release rates in acidic and neutral media. The maximum cumulative release of NPs at 48 h was 84.07 ± 3.79% and 80.25 ± 8.97% at 7.4 and 5.5 pH, respectively (Figure 2).

### 3.3. In Vitro Biological Evaluation of Functionalized NPs

#### 3.3.1. In Vitro Cytotoxicity Assay

**Figure 3:**
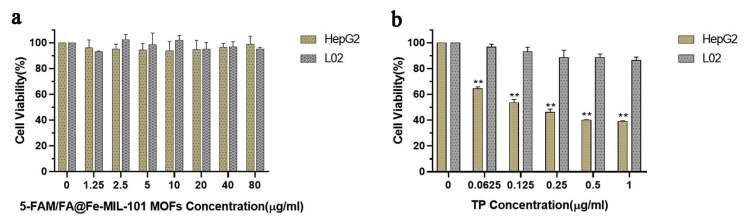
(**a**) The survival rate of HepG2 and HL-7702 cells after 24 h incubation with 5-FAM/FA@Fe-MIL-101; (**b**) effect of 5-FAM/FA/TP@Fe-MIL-101 on the viability of HepG2 and HL-7702 (12 h, the concentrations were measured as TP) (*n* = 3, ** p < 0.01, significantly different compared with control).

To evaluate the safety of 5-FAM/FA@Fe-MIL-101, an MTT experiment was carried out. The results revealed that even at higher concentrations (80 μg/mL), Fe-MIL-101 had no obvious cytotoxicity to HepG2 cells and HL-7702 cells and could be used as a safe nano-drug carrier (Figure 3a). In addition, the toxicity of TP to HepG2 and HL-7702 cells was also evaluated. TP had a strong killing effect on both types of cells, which proved the non-selective toxicity of TP (Figure S5). However, 5-FAM/FA/TP@Fe-MIL-101 had a significantly enhanced cytotoxicity to HepG2 cells and almost no toxicity to HL-7702 cells, indicating that the functional nanoparticles could improve the antitumor activity of triptolide and reduce its toxicity (Figure 3b).

**Figure 4:**
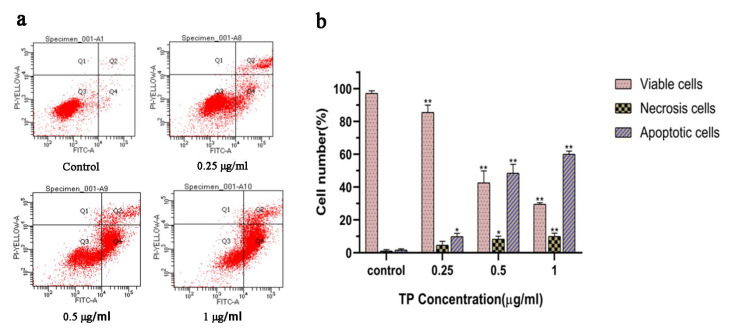
(**a**) Apoptosis detection with Annexin V/PI double staining in HepG2 cells incubated with 5-FAM/FA@TP@Fe-MIL-101 12 h by flow cytometry; (**b**) column bar graph of mean cell florescence for viable, early apoptotic, and late apoptotic cells (*n* = 3, * *p* < 0.05, ** *p* < 0.01, significantly different compared with control).

Annexin V-FITC/PI double staining showed that various concentrations of functionalized NPs could induce the apoptosis of HepG2 cells in a dose-dependent manner (Figure 4a,b). The ROS levels in HepG2 cells were detected using the DCFH-DA fluorescent dye [41]. Compared with the control group, the HepG2 cells incubated with functionalized NPs (TP: 0.5 μg/mL) showed obvious green fluorescence, suggesting that the apoptosis of tumor cells induced by nanoparticles might be related to the damage of the cell redox state (Figure S6). In addition, the mitochondrial membrane potential decreased significantly with the increase in the concentration of functionalized nanoparticles, presenting a significant dose-dependent relationship (Figure S7).

#### 3.3.2. Cell Uptake Study

**Figure 5:**
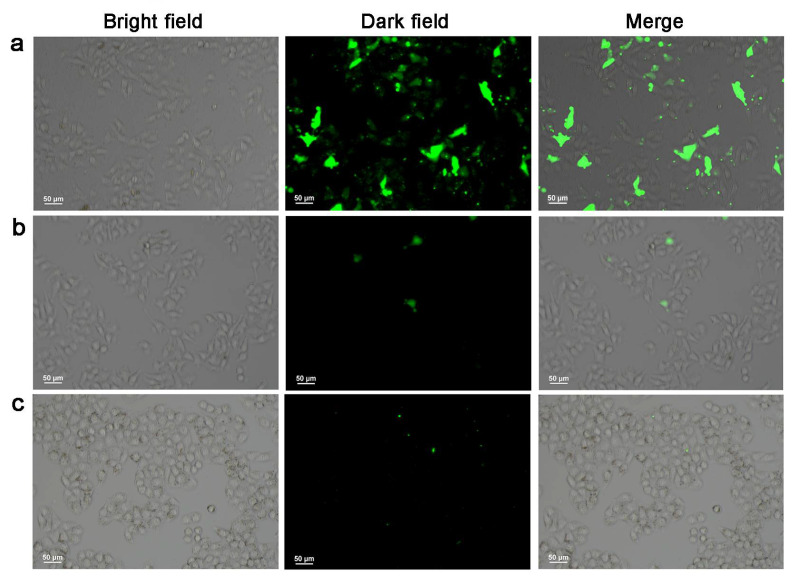
Fluorescence imaging of HepG2 cells cultured with 5-FAM/FA/TP@Fe-MIL-101 (**a**) and 5-FAM/TP@Fe-MIL-101 (**b**) and HL-7702 cells cultured with 5-FAM/FA/TP@Fe-MIL-101 (**c**) for 4 h (exciting light wavelength: 480 nm, magnification: ×200, *n* = 3).

The specific uptake of NPs by tumor cells is key to the effect of antitumor drugs. A laser confocal microscope was used to observe the specific uptake of NPs in vitro. As shown in Figure 5a, when HepG2 cells were cultured with 5-FAM/FA/TP@Fe-MIL-101, green fluorescence was visible in the cells. There was no obvious fluorescence in HepG2 cells treated with 5-FAM/TP@Fe-MIL-101 (Figure 5b) and HL-7702 cells treated with 5-FAM/FA/TP@Fe-MIL-101 (Figure 5c). These results indicated that FA-modified NPs had a high affinity to HepG2, and were preferentially internalized by HepG2 cells through FA-receptor-mediated endocytosis. Dong et al. successfully constructed tumor-targeted NMOF drug carriers by anchoring FA on NMOFs, which enhanced the specific uptake of tumor cells and was an efficient targeted drug delivery system [42].

#### 3.3.3. Western Blot Analysis

**Figure 6:**
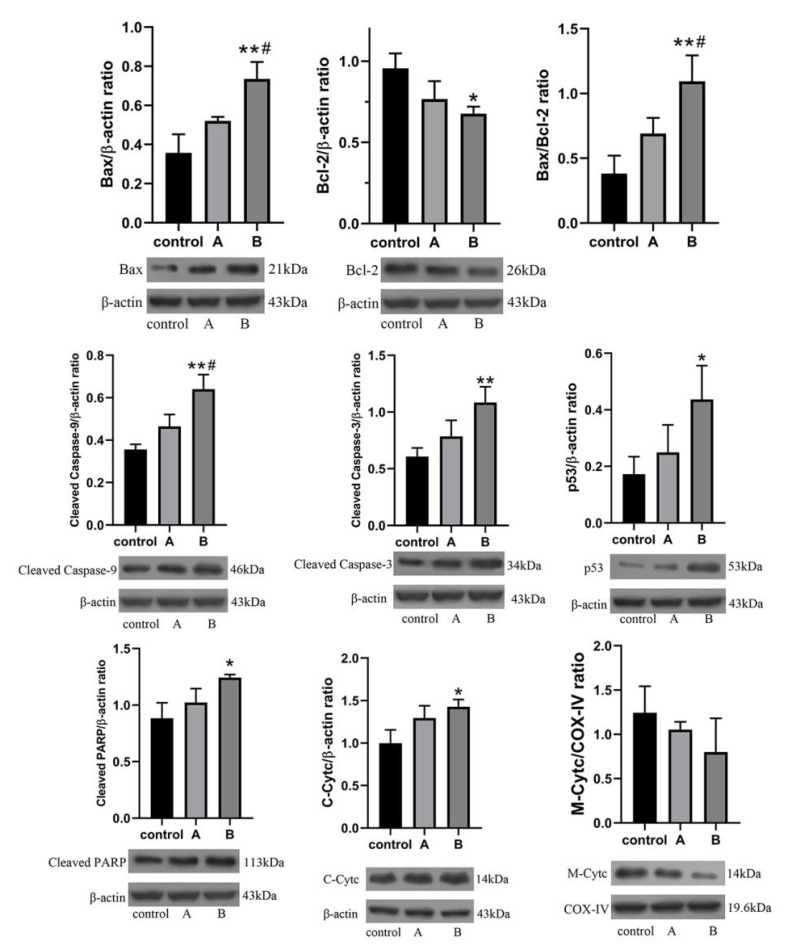
HepG2 cells cultured with (A) TP; (B) 5-FAM/FA/TP@Fe-MIL-101 (* *p* < 0.05, ** *p* < 0.01, significantly different compared with control, # *p* < 0.05, significantly different compared with A group, *n* = 3. Bax and p53, Bcl-2 and PARP used the same β-actin control, respectively).

Western blot results showed that protein expressions of Bax, cleaved Caspase-3, cleaved Caspase-9, p53, and PARP were significantly increased after 12 h treatment of HepG2 cells with TP and functionalized NPs, whereas the expression level of Bcl-2 was significantly decreased (Figure 6). This led to an increase in the Bax/Bcl-2 ratio. The levels of cytochrome c (Cytc) in the cytoplasm were significantly increased, whereas the level of Cytc in mitochondria was significantly decreased, indicating that TP could lead to mitochondrial dysfunction by increasing the permeability of the mitochondrial membrane of HepG2, thereby inducing cell apoptosis [43].

Bax and Bcl-2 are typical apoptosis-related proteins [44,45], and the ratio of Bax/Bcl-2 is affected by ROS, which leads to the increased permeability of mitochondrial membranes and promotes the release of Cytc. Excessive production of ROS can lead to DNA damage [46]. The significantly increased ROS level in HepG2 indicated the existence of an oxidative stress response, which was also confirmed by the increased expression level of the p53 protein [47]. In summary, TP mainly induced the apoptosis of HepG2 through the mitochondrial endogenous pathway. Moreover, the effect of the functionalized NPs group on apoptotic proteins was better than that of the TP group. It was proven that the synthetic functionalized NPs could significantly improve the anti-tumor efficacy.

### 3.4. Distribution of Functionalized Nanoparticles In Vivo

**Figure 7:**
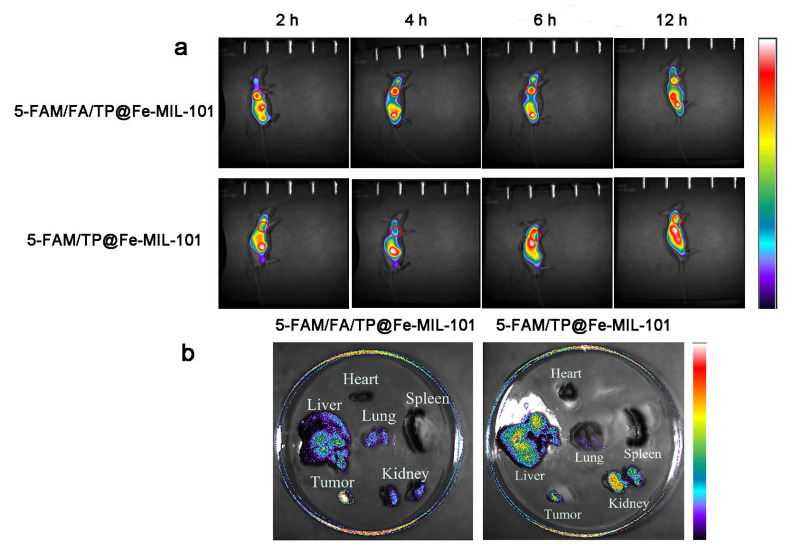
(**a**) Fluorescence imaging was performed in mice at 2 h, 4 h, 6 h, and 12 h after injection NPs; (**b**) fluorescence imaging of major organs and tumors in mice 12 h after NP injection.

The biological distribution of functional nanoparticles in tumor-bearing mice was evaluated by animal imaging in vivo. After 4 h of tail vein injection, the fluorescence intensity of the tumor in the 5-FAM/FA/TP@Fe-MIL-101 group was significantly higher than that in the 5-FAM/TP@Fe-MIL-101 group (Figure 7a). This might be related to the EPR effect and FA-mediated active targeting, which increase the accumulation of NPs at the tumor site [48]. After 12 h of tail vein injection, the tumors and main organs of each group were dissected and analyzed by in vitro fluorescence imaging. The results showed that the functionalized NP group had higher fluorescence intensity in tumor tissues, but lower fluorescence intensity in normal tissues such as the liver and kidney (Figure 7b). Upon modifying the folate ligand on Fe-MIL-101, functional NPs could specifically deliver anticancer drugs to tumors.

### 3.5. Antitumor Effect of Functionalized Nanoparticles In Vivo

**Figure 8:**
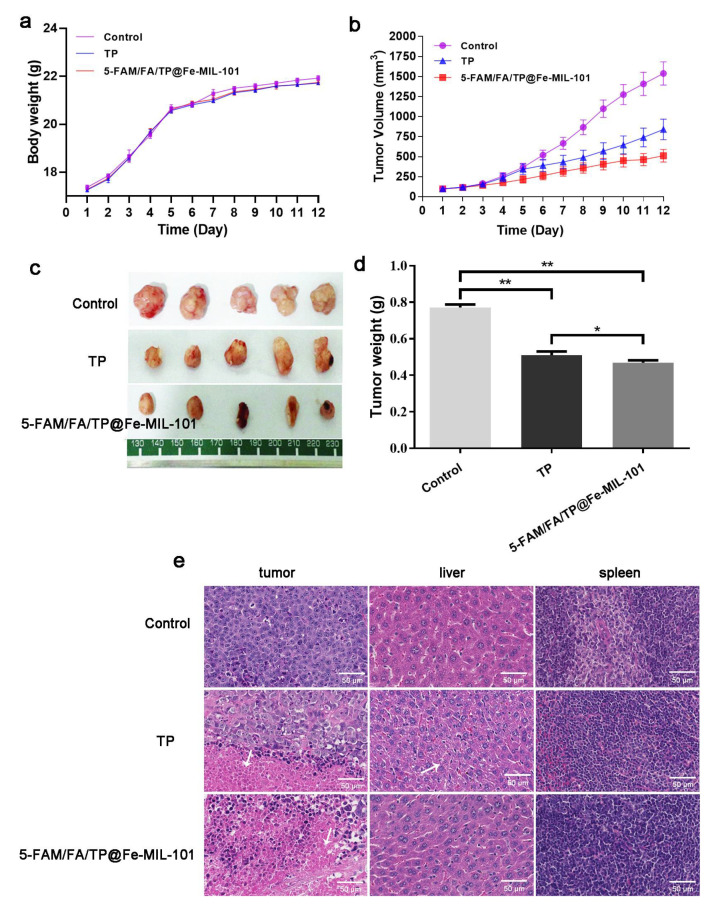
(**a**) Effects of each group on the body weight of nude mice; (**b**) effect of each group on tumor volume in nude mice; (**c**) effect of each group on tumor weight in nude mice; (**d**) tumors in nude mice in each group after administration (* *p* < 0.05, ** *p* < 0.01, significantly different compared with control); (**e**) H&E staining results of the tumor, liver, and spleen in each group (magnification: ×200).

The anti-cancer effect of functionalized NPs was further evaluated via tail vein injection at a dose of 0.2 mg/kg. After 12 days of administration, there was no significant difference in body weight between the different treatment groups and the control group, indicating that NPs did not increase systemic toxicity compared to direct TP administration (Figure 8a). There were significant differences in tumor volume between the groups. The average tumor volume in the saline group was 1538.50 ± 461.75 mm^3^; in the TP group it was 841.38 ± 406.63 mm^3^; and in the 5-FAM/FA/TP@Fe-MIL-101 group it was 511.76 ± 250.79 mm^3^ (Figure 8b,c). Similarly, the tumor masses of the TP group and the 5-FAM/FA/TP@Fe-MIL-101 group were smaller than those of the saline group (Figure 8d). Compared with the control group and pure TP, 5-FAM/FA/TP@Fe-MIL-101 exhibited a superior anticancer effect, which might be related to the EPR effect of NPs and the active targeting effect mediated by FA. Histopathological results confirmed that tumors in the saline group were scattered in the liver, hepatocytes in the TP group had inflammation, and the functionalized nanoparticle group had no obvious side effects on the main organs (liver, spleen) of mice (Figure 8e). TUNEL and DAPI staining (Figure S8) showed that the fluorescence intensity of the TP group and the functionalized NPs group was significantly increased, indicating a significant increase in the percentage of tumor cell apoptosis. The antitumor effect of functionalized NPs was more obvious, which proved that this new drug delivery system had better anti-tumor efficacy compared with TP direct administration.

### 3.6. Safety Evaluation of Functionalized NPs In Vivo

After 14 days of continuous administration, compared with the control group, the functionalized nanoparticle group had no significant effects on ALT, AST, ALP, or BUN (Figure S9a). In addition, histopathological results showed that no abnormalities were found in the liver, spleen, or kidney tissues of mice in the functionalized NPs group, indicating that the prepared functionalized NPs have good biological safety (Figure S9b).

## 4. Conclusions

In this research, we constructed a new multifunctional drug carrier, 5-FAM/FA/TP@Fe-MIL-101, to enhance antitumor cytotoxicity for cancer therapy. In vitro drug release results showed that TP exhibited good drug release performance in PBS. Cytotoxicity experimental results indicated that NPs have good antitumor activity. Through fluorescence imaging and flow cytometry analysis, NPs demonstrated good folate targeting properties. 5-FAM/FA/TP@Fe-MIL-101 has a strong tumor suppression rate in nude mice with liver cancer and possesses good antitumor effect. In conclusion, a drug delivery system based on MOFs with tumor targeting and fluorescence imaging was designed and constructed, providing new ideas for targeted drug delivery.

## Data Availability

The data presented in this study are available on request from the cerresponding authors.

## Supplementary Materials

The following are available online at https://www.mdpi.com/article/10.3390/pharmaceutics13111945/s1, Figure S1: XRD pattern of Fe-MIL-101 (a: different solvent amount, b: different reaction time, c: different reaction tempera-ture, d: different ratio), Figure S2: SEM (a), TEM (b) and DLS (c) images of Fe-MIL-101 (the scale bar indicated 100 nm), Figure S3: (a) XRD patterns of Fe-MIL-101, (b) N2 adsorptio-desorp-tion isotherms of Fe-MIL-101, Figure S4: FTIR spectra of Fe-MIL-101 (A), FA (B), 5-FAM (C), 5-FAM/FA@Fe-MIL-101 (D), Figure S5: (a) Effect of TP on the viability of HepG2; (b) Effect of TP on the viability of L02, Figure S6: (a) Fluorescence microscope was used to observe control group and TP group; (b) Analysis of ROS generation using the oxidant sensitive fluorescent probe DCFH-DA; (c) Column bar graph of mean cell florescence for DCFH-DA. (* *p* < 0.05, ** *p* < 0.01), Figure S7: (a) Fluorescence microscope was used to observe control group and TP group (Red represents normal mitochondria, whereas green represents depolarized mitochondria); (b) MMP detection with JC-1 staining in different groups with flow cytometry flow cytometry. (Blue repre-sents normal mitochondria, whereas green represents depolarized mitochondria); (c) Column bar graph of mean cell florescence for JC-1. (** *p* < 0.01), Figure S8: (a) TNUEL and DAPI double staining images of tumor tissues in each group (magnification: ×200); (b) Percentage of cell apop-tosis in tumor tissues of each group (Group1:5-FAM/FA/TP@Fe-MIL-101, Group2:TP; * *p* ≤ 0.05, ** *p* ≤ 0.01), Figure S9: (a) ALT, AST, ALP, BUN indexes were detected; (b) H&E staining results of liver, spleen and kidney in each group (magnification: ×200).
