# Supplementary material for: Construction of a Multifunctional Nano-Scale Metal-Organic Framework-Based Drug Delivery System for Targeted Cancer Therapy
Source: Pharmaceutics. 2021 Nov 17;13(11):1945. doi: 10.3390/pharmaceutics13111945 (PMC8619429; doi:10.3390/pharmaceutics13111945)
Supplement: Supplementary file 1 [file pharmaceutics-13-01945-s001.zip › pharmaceutics-1433709-supplementary.pdf]

pharmaceutics

Supplementary Materials: Construction of a Multifunctional Nano-Scale Metal-Organic Framework-Based Drug Delivery System for Targeted Cancer Therapy

Mengru Cai, Yawen Zeng, Manting Liu, Longtai You, Huating Huang, Yang Hao, Xingbin Yin, Changhai Qu, Jian Ni and Xiaoxv Dong

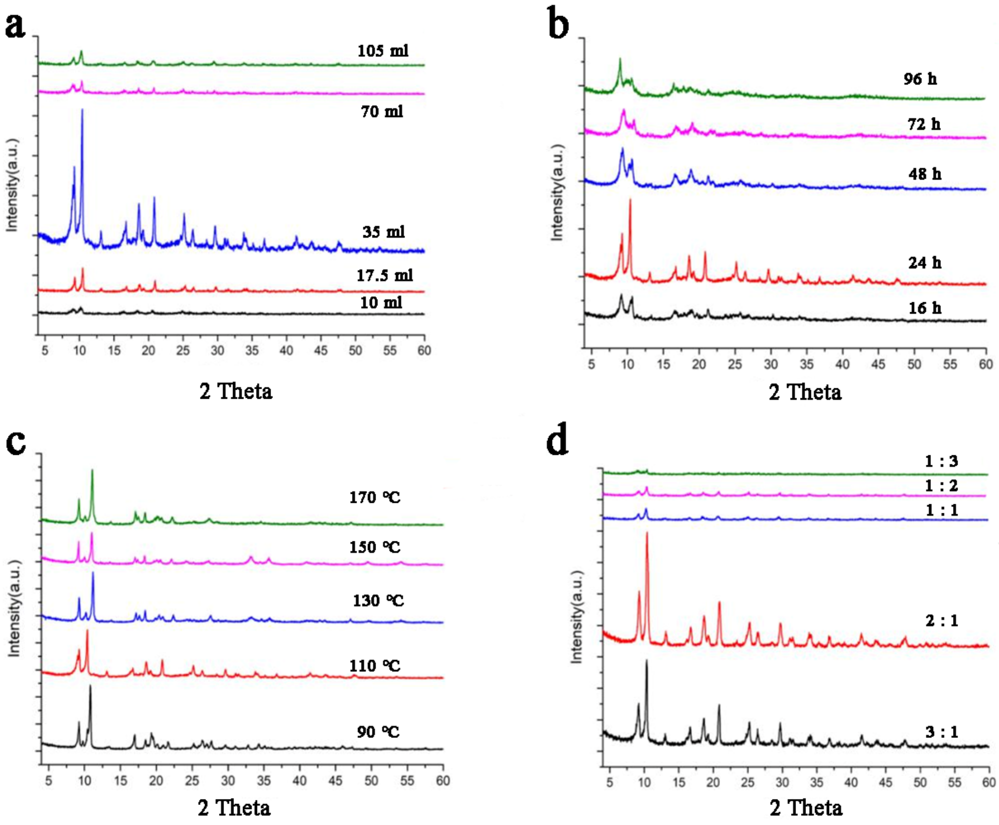

Figure S1. XRD pattern of Fe-MIL-101 (**a**: different solvent amount, **b**: different reaction time, **c**: different reaction temperature, **d**: different ratio).

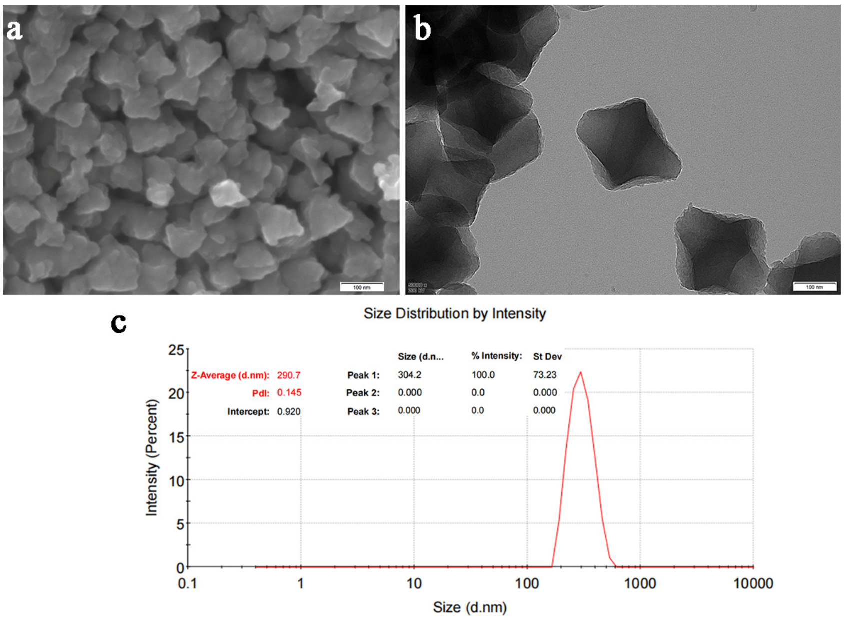

Figure S2. SEM (**a**), TEM (**b**) and DLS (**c**) images of Fe-MIL-101 (the scale bar indicated 100 nm).

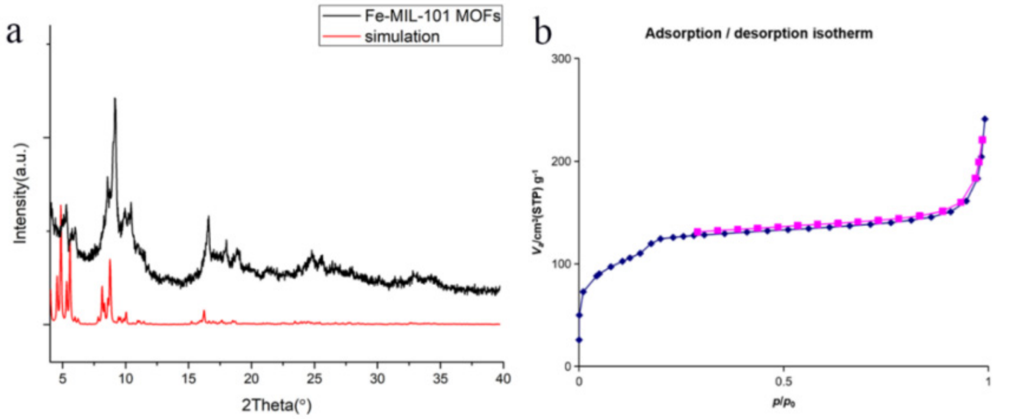

Figure S3. (**a**) XRD patterns of Fe-MIL-101, (**b**) N_2 adsorptio-desorp-tion isotherms of Fe-MIL-101.

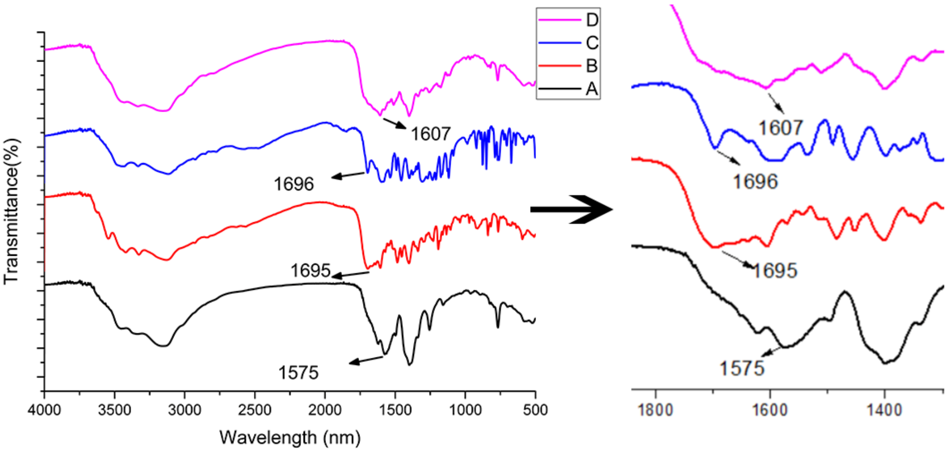

Figure S4. FTIR spectra of Fe-MIL-101 (A), FA (B), 5-FAM (C), 5-FAM/FA@Fe-MIL-101 (D).

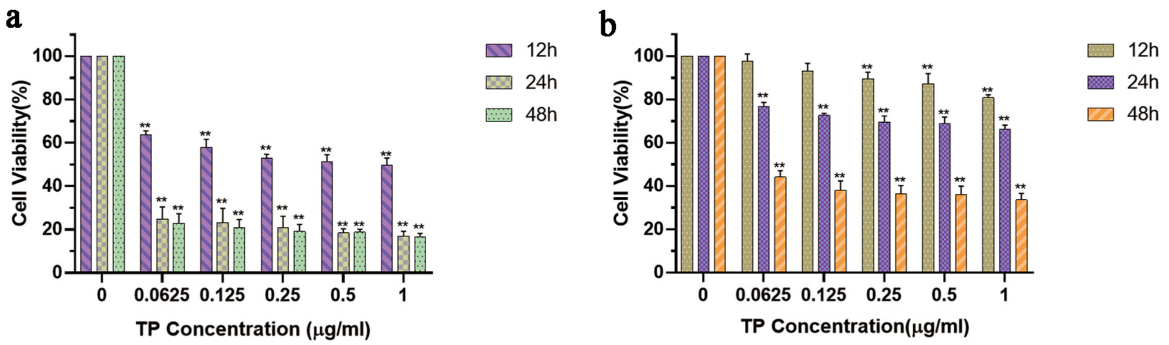

Figure S5. (**a**) Effect of TP on the viability of HepG2; (**b**) Effect of TP on the viability of L02. ($n = 3$, $^{**}p < 0.01$, significantly different compared with control).

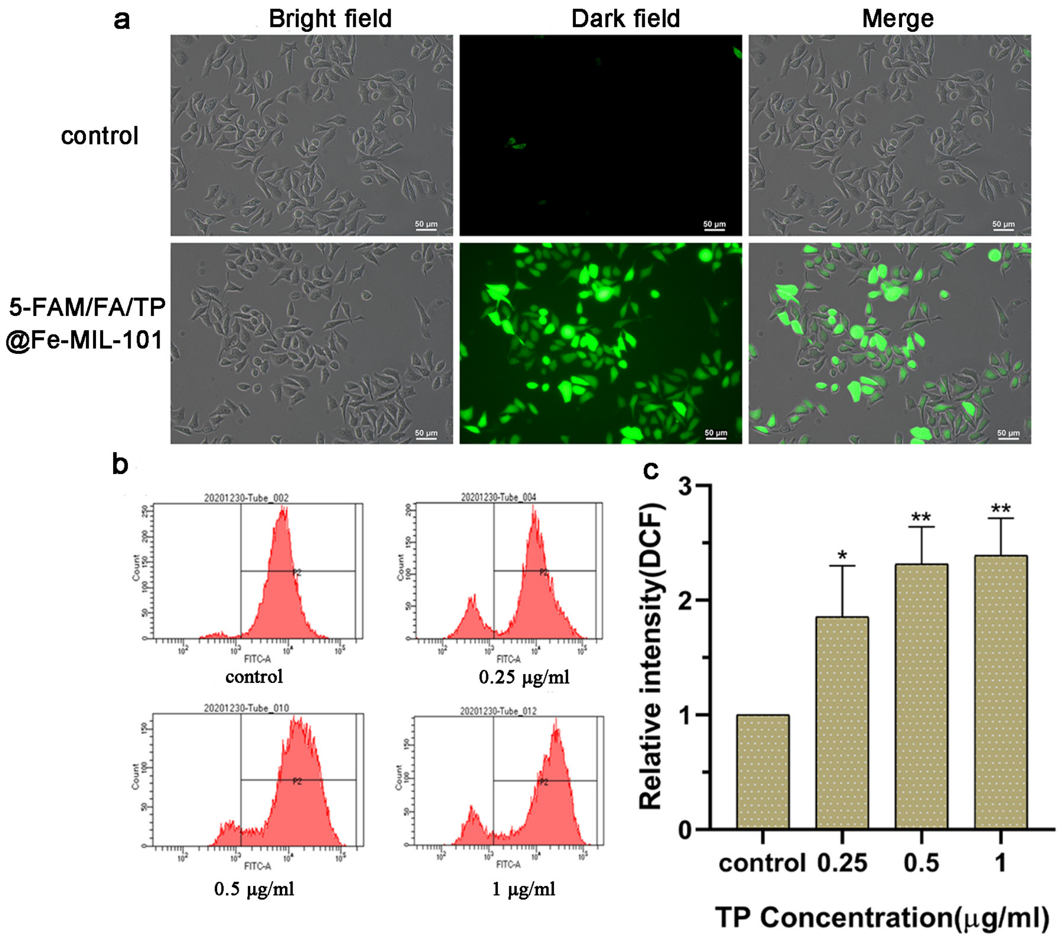

Figure S6. (**a**) Fluorescence microscope was used to observe control group and TP group; (**b**) Analysis of ROS generation using the oxidant sensitive fluorescent probe DCFH-DA; (**c**) Column bar graph of mean cell florescence for DCFH-DA. ($^{*}p < 0.05$, $^{**}p < 0.01$).

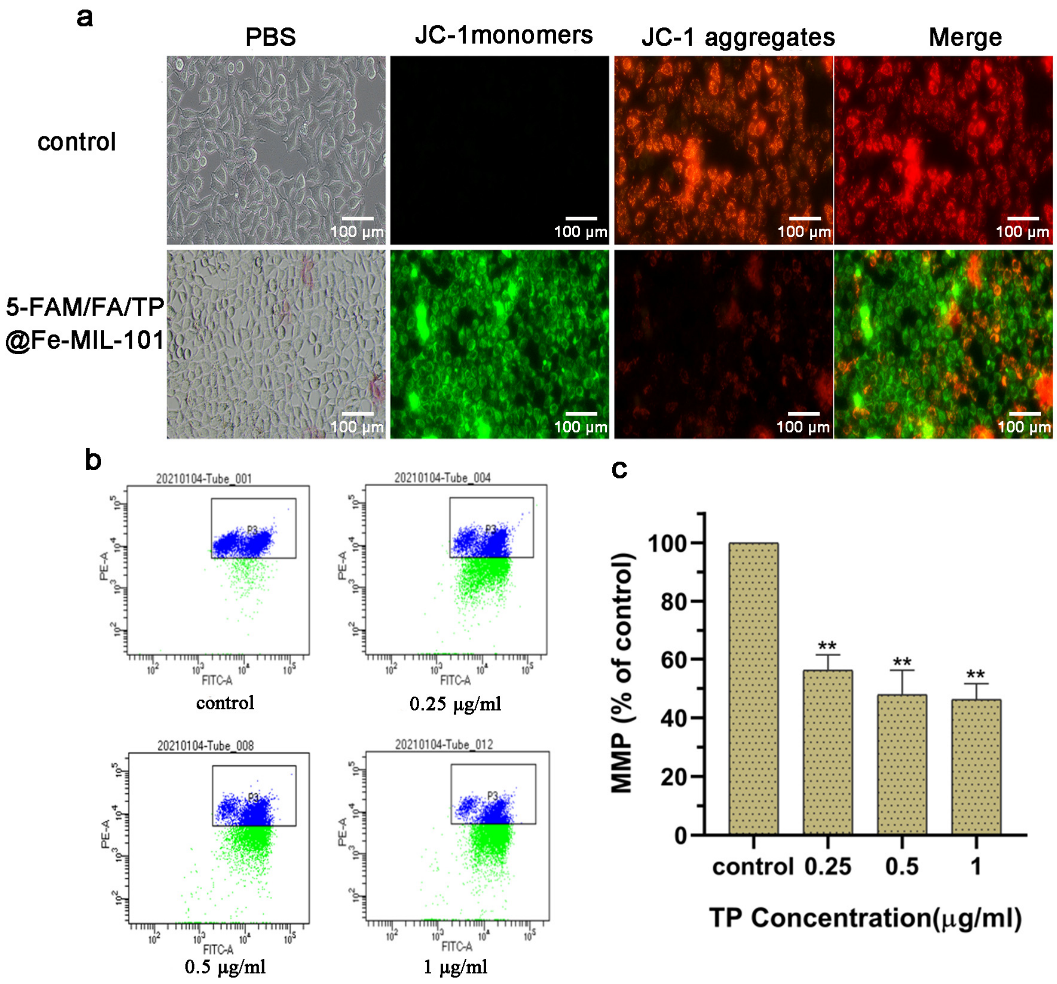

Figure S7. (**a**) Fluorescence microscope was used to observe control group and TP group (Red represents normal mitochondria, whereas green represents depolarized mitochondria); (**b**) MMP detection with JC-1 staining in different groups with flow cytometry flow cytometry. (Blue represents normal mitochondria, whereas green represents depolarized mitochondria); (**c**) Column bar graph of mean cell florescence for JC-1. (**p < 0.01).

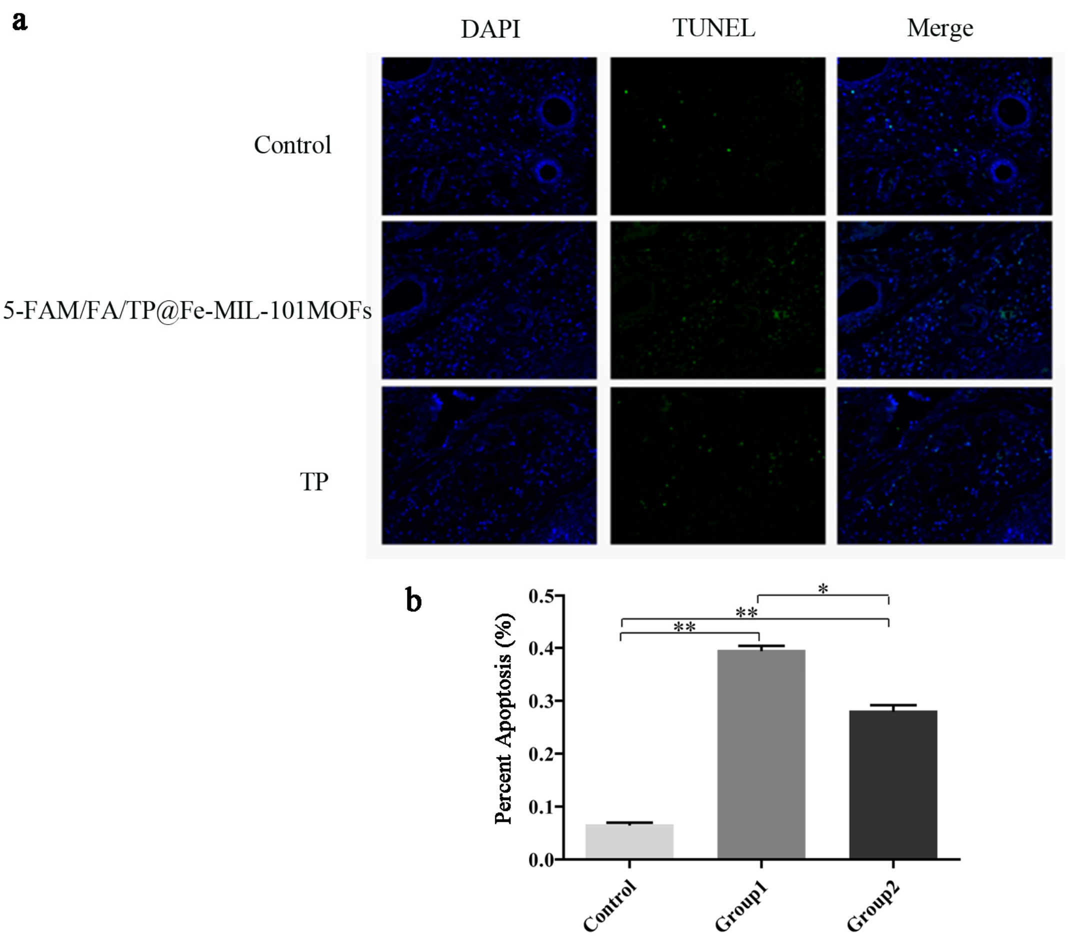

Figure S8. (**a**) TNUEL and DAPI double staining images of tumor tissues in each group (magnification: ×200); (**b**) Percentage of cell apoptosis in tumor tissues of each group（Group1:5-FAM/FA/TP@Fe-MIL-101, Group2:TP; *$p \leq 0.05$, **$p \leq 0.01$).

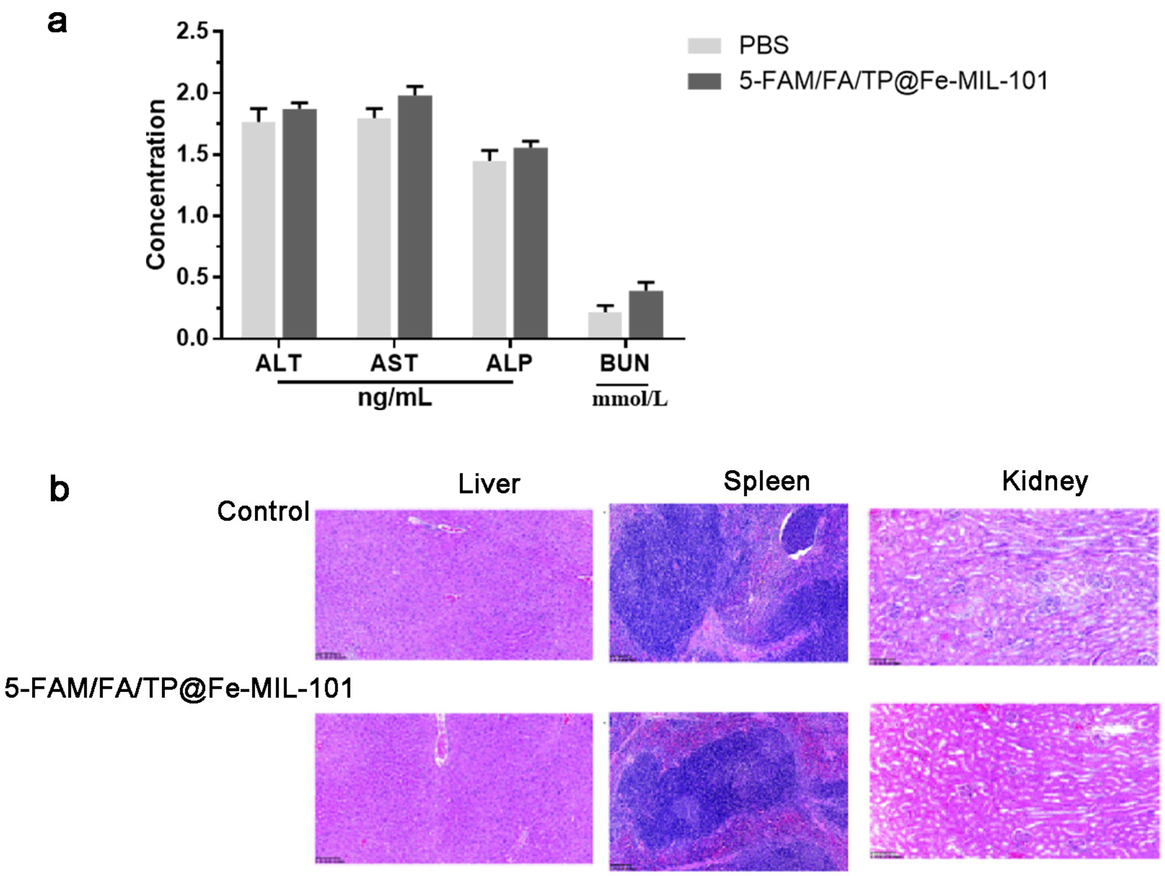

Figure S9. (**a**) ALT, AST, ALP, BUN indexes were detected; (**b**) H&E staining results of liver, spleen and kidney in each group (magnification: ×200).
